# Gross Tumor Volume Definition and Comparative Assessment for Esophageal Squamous Cell Carcinoma From 3D ^18^F-FDG PET/CT by Deep Learning-Based Method

**DOI:** 10.3389/fonc.2022.799207

**Published:** 2022-03-17

**Authors:** Yaoting Yue, Nan Li, Husnain Shahid, Dongsheng Bi, Xin Liu, Shaoli Song, Dean Ta

**Affiliations:** ^1^ Center for Biomedical Engineering, School of Information Science and Technology, Fudan University, Shanghai, China; ^2^ Department of Nuclear Medicine, Fudan University Shanghai Cancer Center, Shanghai, China; ^3^ Academy for Engineering and Technology, Fudan University, Shanghai, China

**Keywords:** definition, gross tumor volume, esophageal squamous cell carcinoma, deep learning, equivalent truncated elliptical cone, comparative assessment

## Abstract

**Background:**

The accurate definition of gross tumor volume (GTV) of esophageal squamous cell carcinoma (ESCC) can promote precise irradiation field determination, and further achieve the radiotherapy curative effect. This retrospective study is intended to assess the applicability of leveraging deep learning-based method to automatically define the GTV from 3D ^18^F-FDG PET/CT images of patients diagnosed with ESCC.

**Methods:**

We perform experiments on a clinical cohort with 164 ^18^F-FDG PET/CT scans. The state-of-the-art esophageal GTV segmentation deep neural net is first employed to delineate the lesion area on PET/CT images. Afterwards, we propose a novel equivalent truncated elliptical cone integral method (ETECIM) to estimate the GTV value. Indexes of Dice similarity coefficient (DSC), Hausdorff distance (HD), and mean surface distance (MSD) are used to evaluate the segmentation performance. Conformity index (CI), degree of inclusion (DI), and motion vector (MV) are used to assess the differences between predicted and ground truth tumors. Statistical differences in the GTV, DI, and position are also determined.

**Results:**

We perform 4-fold cross-validation for evaluation, reporting the values of DSC, HD, and MSD as 0.72 ± 0.02, 11.87 ± 4.20 mm, and 2.43 ± 0.60 mm (mean ± standard deviation), respectively. Pearson correlations (R^2^) achieve 0.8434, 0.8004, 0.9239, and 0.7119 for each fold cross-validation, and there is no significant difference (*t* = 1.193, *p* = 0.235) between the predicted and ground truth GTVs. For DI, a significant difference is found (*t* = −2.263, *p* = 0.009). For position assessment, there is no significant difference (left-right in x direction: *t* = 0.102, *p* = 0.919, anterior–posterior in y direction: *t* = 0.221, *p* = 0.826, and cranial–caudal in z direction: *t* = 0.569, *p* = 0.570) between the predicted and ground truth GTVs. The median of CI is 0.63, and the gotten MV is small.

**Conclusions:**

The predicted tumors correspond well with the manual ground truth. The proposed GTV estimation approach ETECIM is more precise than the most commonly used voxel volume summation method. The ground truth GTVs can be solved out due to the good linear correlation with the predicted results. Deep learning-based method shows its promising in GTV definition and clinical radiotherapy application.

## Introduction

According to the latest 2020 global cancer statistics, esophageal cancer (EC) ranks seventh and sixth respectively in terms of incidence (3.1%) and mortality rate (5.5%) (1). EC contains 2 most common histologic subtypes: squamous cell carcinoma and adenocarcinoma, of which the esophageal squamous cell carcinoma (ESCC) is relatively sensitive to the radiation rays (1). As a result, radiotherapy is a significant component of comprehensive therapy for ESCC patients. Three steps are included during clinical radiation treatment: CT localization, irradiation field (IF) delineation, and radiotherapy planning. Thereinto, excessive IF is current the major problem, which may cause radiation injury of lungs, pneumonia, oesophagitis, etc. A main reason for the excessive IF lies in the inaccurate definition of target volume, which current relies on the manual way, not only exhausting radiologists on a treadmill but also lacking consensus due to the high inter- and intra-observer variability (2, 3). Thus, the precise definition of target volume is vital for curative treatment. From this point of view, this work is aimed at leveraging artificial intelligence-based method to explore accurate definition of target volume, as an assistant to help clinicians to determine more precise IF.

Target volume definition involves the accurate delineation and prediction of the gross tumor volume (GTV) on medical images (4). For one thing, once the GTV is established, under the consideration of involved metastasis lymph nodes and organs at risk, the clinical target volume will be defined by expanding and measuring the adjacent sub-clinical disease margins (2, 5). Further, the clinical target volume plus a margin gives the planning target volume (6). Thus, the precise knowledge of GTV can assist to maximize the therapy to the target lesion while minimizing damage to the surrounding normal organs or tissues (7). For another, other metabolic metrics with potential prognostic value can be derived from the GTV like the total lesion glycolysis and total tumor surface ratio (8). Meanwhile, GTV has been demonstrated as an important prognostic determinant for ESCC patients (9, 10), and the research of Dubben et al. suggested that individual tumor volume should be reported in clinical studies and considered in data analyses (11). Currently, Fluorine 18-fluorodeoxyglucose positron emission tomography/computed tomography (^18^F-FDG PET/CT) guided precise radiotherapy for EC patients play an important role (12, 13), as this multi-modality imaging technique simultaneously provides both the metabolic and anatomical information which are complimentary to determine and correct the GTV (14–16). Based on this, we retrospectively analyze an annotated clinical 3D ^18^F-FDG PET/CT image set of 164 patients diagnosed with ESCC, for the purpose of assessing the feasibility of automatically defining GTV by artificial intelligence-based method.

At present, for the definition of GTV, advances in GTV delineation for EC *via* deep learning methods are showing promise (2, 17–20), but there has been limited researches into the GTV estimation. Given that, we put more effort on the estimation part. The old-fashioned method is a cuboid structure, which first needs to determine six furthest points in the main six dimensions of the tumor (4). As most tumors grow likes a sphere or spheroid, the cuboid structure will contain extra normal tissues which should not be irradiated (4). After that, the spherical shape produced from conformal planning is considered (4). In the year 2006, Crehange et al. took the tumor as two opposing truncated cones, and presented a volumetric assessment method (10). Though these rough approximations get closer and closer to the target shape, there is a certain error. The current most common method for GTV estimation is to compute the sum of lesion voxel volumes in the medical images (21, 22). But since the tumor marginal area does not fill the pixel grids, the predicted GTV by this method is actually bigger than the true value. According to a recent study, equivalent ellipse can get a good fitting of elliptical or circular aggregate particles (23). This motivates us to use equivalent ellipse to fit lesion area on the axial slice. Next, inspired by the volumetric assessment method of Crehange et al., we take the volumetric tumor between two adjacent slices as a truncated elliptical cone, and then combine the integral technique to estimate the GTV value. By this way, the estimated GTV will get closer to the actual value than the voxel volume summation method, which includes the extra volume capacity in the corners of the cuboid voxel.

Before the GTV estimation, it requires the lesion segmentation step from the ^18^F-FDG PET/CT images. To achieve this, we employ the state-of-the-art (SOTA) esophageal GTV segmentation network, progressive semantically-nested network (PSNN), to delineate the tumor regions (2). So, to summarize the whole process, we first leverage the SOTA esophageal GTV segmentation network PSNN to implement the delineation work. Afterwards, the newly proposed ETECIM is used to estimate the GTV value. Last, we perform statistical analyses by using the SPSS software package to make a comparative assessment, for the purpose of evaluating the applicability of deep learning-based method to automatically define the GTV from 3D ^18^F-FDG PET/CT images of patients diagnosed with ESCC.

## Material and Methods

### Data Acquisition and Ground Truth Generation

This retrospective study was approved by the Ethics Committee of Fudan University Shanghai Cancer Center (No. 1909207-14-1910). The requirement of written informed consent was waived, and the data were analyzed anonymously. We collected 166 ESCC patients enrolled between February 2014 and September 2019 from the Fudan University Shanghai Cancer Center. All the ^18^F-FDG PET/CT scans of patients were performed by a whole-body PET/CT scanner (Siemens Biograph mCT Flow PET/CT). In a state of fasting (at least 6 h), all the patients received a glucose level test and the blood glucose levels should be less than 10 mmol/L. The whole-body ^18^F-FDG PET/CT acquisitions were started 1 h after the intravenous injection of ^18^F-FDG (7.4 MBq/kg). For the Siemens Biograph mCT Flow PET/CT scanner, a spiral CT scan with the protocol (120 kV, 140 mA, 5 mm slice thickness) was conducted. The followed PET scan lasted 2–3 min per bed position, with PET images being reconstructed iteratively *via* CT data for attenuation correction. The final obtained PET/CT images were clearly displayed and were available in DICOM format.

DICOM files of the ^18^F-FDG PET/CT data were imported to ITK-SNAP software (Version 3.6, United States), and the ground truth GTVs were delineated by 2 experienced nuclear medicine physicians on the CT axial slices with referring to the corresponding PET images. After that, a chief physician with rich clinical experience over 15 years reviewed and determined the final ground truth mask. The delineation follows the standards for an esophageal wall thickness >5 mm or an esophageal wall diameter (without gas) >10 mm.

The inclusion criteria followed principles (1): pathologically confirmed esophageal squamous cell cancer (2); complete and available ^18^F-FDG PET/CT scan data before RT therapy (3); complete and available manual delineation for each ^18^F-FDG PET/CT data. Thereafter, 2 patients were excluded for the lack of integrity on ground truth GTV. Hence, a total of 164 patients were finally included in the study population. To ensure rationality of the experiments, this study performs 4-fold cross-validation for evaluation.

### Data Pre-Processing

The reconstructed CT scans are with two spatial resolutions of 0.98 × 0.98 × 5 mm^3^ and 1.37 × 1.37 × 5 mm^3^, and the reconstructed PET scans are with 4.06 × 4.06 × 5 mm^3^ and 4.07 × 4.07 × 5 mm^3^. For all CT slices, the matrix size is 512 × 512, whereas the PET slices have two types 200 × 200 and 168 × 168. Thus, all PET slices were up-sampled in the axial plane, leading to the size of 512 × 512 *via* the bicubic interpolation algorithm (24). The reason that we choose the bicubic interpolation algorithm for interpolation lies in its advantage of conserving detailed information, which is vital in the segmentation step. As for the spatial resolution, we remain its diversity unchanged to enhance robustness of the segmentation network. Next, to improve the contrast between lesion area and surrounding soft tissue in CT images, pixel values outside of −150 to 150 were set to −150 and 150. Then PET and CT images were all normalized to the interval of [0, 1]. Last, though PET/CT images had been registered by the hardware of the PET/CT scanner (Siemens Biograph mCT Flow PET/CT), there is slight deviation caused by involuntary respiratory movement of the patient during the image acquisition process. As the focus of this work is not on the registration, here we simply use the multi-mode intensity registration algorithm to correct the deviation (25).

### Segmentation Model and Training

After pre-processing, the obtained dual-modality images (PET and CT) were used to conduct the automatic segmentation of esophageal GTV based on the deep network PSNN (2). Jin et al. reversed the direction of deeply-supervised pathways in the progressive holistically-nested network (26), and then combined the structure of U-Net (27) to design a novel PSNN architecture (2). They have demonstrated that their proposed parameter-less PSNN could progressively aggregate the higher-level semantic features down to lower-level space in a deeply-supervised way, achieving the SOTA segmentation performance for esophageal GTV. Hence, this work followed the setup described in (2) to build the PSNN model for the GTV auto-segmentation task. For training, data cropping was first conducted. Due to the low occupancy of esophageal carcinoma in PET/CT images, it was necessary to crop each PET/CT volume scan to a region of interest to alleviate both the class imbalance issue and storage limit. Afterwards, we set the algorithm to randomly extract 16 training patches of size 64 × 64 × 64 from each region of interest and performed one of the data augmentations (rotate 90°, or flip left and right, or flip up and down, or flip lift and right first and then rotate 90°, or remain unchanged). The number of training volumes was 16 times increase after the data augmentation. The training was performed on a Windows server equipped with Nvidia GeForce GT 710 graphical processing units. The Adam Optimizer with an initial learning rate 10^–2^ (reduced by 0.95 every 5 epochs) was applied to the gradient descent optimization.

### GTV Estimation Based on ETECIM

**Figure 1:**
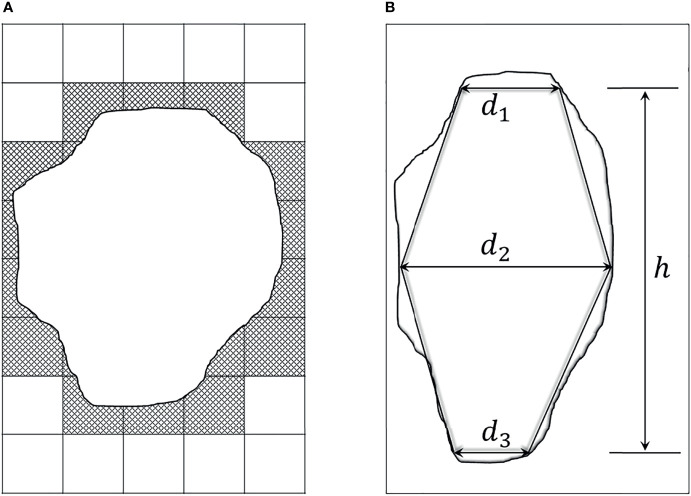
Two existing methods for GTV estimation. **(A)** A cross-section view of the voxel volume summation method. The middle white part denotes the lesion mask, and the surrounding dash area denotes the extra computation. **(B)** Two opposed truncated cones summation method, *d*_1_ and *d*_3_ respectively represent the cranial and caudal transverse diameters of the tumor. *d*_2_ is the maximal transverse diameter, and *h* is the tumor height.

The commonly used method for GTV prediction is to compute the sum of lesion voxel volumes (21, 22). But since the tumor marginal area does not fill the voxel grids, the predicted GTV by this method is bigger than the actual value. As shown in **Figure 1A**, a cross-section view of this voxel volume summation method, the lesion mask is the middle white part, whereas the predicted area *via* computing the sum of pixels will extra cover the hatched section. Therefore, estimated GTV by this method will extra include the volume capacities in the corners of the cuboid voxels.

According to a recent study, equivalent ellipse can get a good fitting of elliptical or circular aggregate particles (23). This motivates us to apply equivalent ellipse to fit lesion area, and adopt a geometric approach to estimate GTV value for avoiding the shortcoming of voxel volume summation method. To be specific, inspired by the method of Crehange et al. which roughly considered the tumor as two opposing truncated cones (**Figure 1B**) (10), we deem the volumetric tumor between two adjacent slices as a truncated elliptical cone, and then take the integral technique to estimate GTV value. The detailed introduction of this proposed method is described as follows.

Suppose that the foreground of the binarized ground truth or predicted mask is a system of *N* mass points. Due to the same gray value of each point, we assume that they have the unit quality, with coordinates from (*x*_1_, *y*_1_), (*x*_2_, *y*_2_),…, to (*x_N_*, *y_N_*). Besides, we assume that a line, denoted as L, passes through the origin coordinates (0, 0). As the foreground (arbitrarily shaped lesion) in the binarized ground truth or predicted mask can be considered as a planar rigid, the moment of inertia of the foreground rotating about line L is defined,


(1) $$I=\Sigma_{i=1}^{N}\;d_{i}^{2},$$

where *d_i_* is the vertical distance from point (*x_i_*, *y_i_*) to line L. Suppose that the two direction cosines of line L are *α* and *β*, respectively, then formula (1) can be rewritten as,


(2) $$I=I_{x}\alpha^{2}+I_{y}\beta^{2}-2I_{xy}\alpha \beta ,$$

where $I_{x}=\Sigma_{i=1}^{N}\;x_{i}^{2},\;I_{y}=\Sigma_{i=1}^{N}\;y_{i}^{2}$ , denoting the moments of inertia of the foreground rotating about the X-axis and Y-axis. $I_{xy}=\Sigma_{i=1}^{N}\;x_{i}y_{i}$ , denoting the inertia product.

Formula (2) will be interpreted in a simple geometric way. We know that a second-order curve *C* with its center at the origin of coordinates can be expressed as,


(3) $$Ax^{2}+By^{2}-2Hxy=1,$$

where *A*, *B*, *H* and *C* are constants. If using *r* to represent the vector from the origin to the curve, with the cosines are α and β, we get *x* = *r*α and *y* = *r*β. Then, formula (3) can be rewritten as,


(4) $$r^{2}(A\alpha^{2}+B\beta^{2}-2H\alpha \beta )=1.$$

Refer to formula (2), if setting *A* = *I_x_*, *B* = *I_y_*, *and H* = *I_xy_*, formula (4) is equivalent to,


(5) $$r^{2}(I_{x}\alpha^{2}+I_{y}\beta^{2}-2I_{xy}\alpha \beta )=r^{2}I=1.$$

As the moment of inertia *I* is always greater than zero, *r* must be a finite value, that is to say, the second-order curve *C* is closed. Therefore, *C* must be an ellipse, which is called inertia ellipse. Hence, according to the moments of inertia of the foreground, a corresponding inertia ellipse will be obtained to simulate the distribution of pixels in the foreground. Due to the foreground and its inertia ellipse approximately have the same area, the inertia ellipse is also called the equivalent ellipse of the foreground (28). The orientations of the two principal axes of the equivalent ellipse can be calculated *via* solving the eigenvalues of the second-order curve *C*. Let *k* and *l* denote the slopes of two principal axes, respectively, then *k* and *l* are defined as follows,


(5) $$k=\frac{(A-B)+\sqrt{{\left(A-B\right)}^{2}+4H^{2}}}{2H},$$

(6) $$l=\frac{(A-B)-\sqrt{{\left(A-B\right)}^{2}+4H^{2}}}{2H}.$$

Let φ_1_ and φ_2_ respectively represent the sharp angles between the long and short principal axes and the positive X-axis, we can get φ_1_ = *arctan*(–*k*), and φ_2_ = *arctan*(–*l*). Accordingly, we can use the approximate area *M* (the number of all the pixels in the foreground multiplied by the unit pixel area) of the equivalent ellipse to calculate the half-lengths of the two principal axes as,


(7) $$a=\sqrt{\frac{2[(A+B)+\sqrt{{\left(A-B\right)}^{2}+4H^{2}}]}{M},}$$

(8) $$b=\sqrt{\frac{2[(A+B)-\sqrt{{\left(A-B\right)}^{2}+4H^{2}}]}{M}.}$$

**Figure 2:**
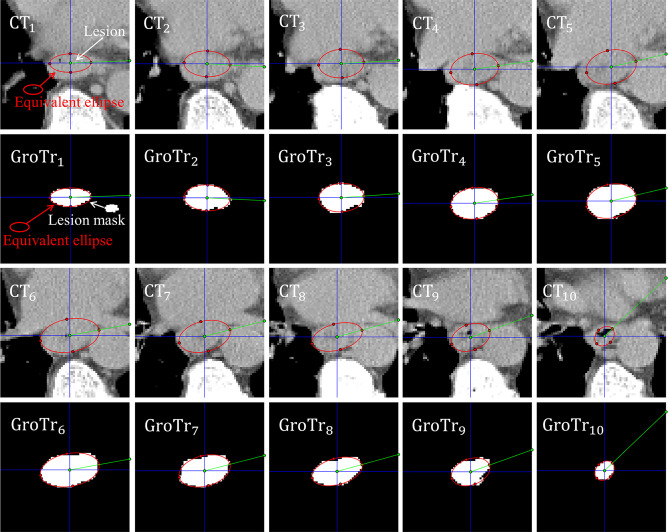
Esophageal carcinoma approximately assessed by its corresponding equivalent ellipses. *CT_i_* and *GroTr_i_* (*i* = 1,2…, 10) denote the *i th* CT slice and its corresponding ground truth mask of tumor. The red ellipse is the equivalent ellipse of lesion. The white mask is the lesion mask. The intersection angles between the green line segment and the blue horizontal straightness represent the sharp angles between the long principal axes and the positive X-axis.

As depicted in **Figure 2**, the esophageal carcinoma of a patient can be approximately assessed by the corresponding equivalent ellipses.

**Figure 2** shows that the equivalent ellipses accurately simulate the distribution of tumor pixels. Besides, for the adjacent slices, the sharp angles between the long principal axes and the positive X-axis (denoted by the intersection angles between the green line segment and the blue horizontal straightness) are not moving much. Therefore, we take the tumor volume between two adjacent slices as the volume of an equivalent truncated elliptical cone, and sum all the equivalent volumes of adjacent slices to get the final GTV estimate. For the sake of brevity, we call this proposed GTV prediction method as equivalent truncated elliptical cone integral method (ETECIM), which is defined as,


(9) $$GTV=\Sigma_{i=m}^{n-1}\frac{\pi h}{6}(2(a_{i}b_{i}+a_{i+1}b_{i+1})+a_{i}b_{i+1}+b_{i}a_{i+1}),$$

where *m* and *n* respectively denote the sequence numbers of the cranial and caudal slices of the tumor. *h* is the axial resolution. *a_i_* and *b_i_* represent the half-length of the long and short principal axes for the equivalent ellipse in the *i th* slice. (π*h*/6)(2(*a_i_b_i_* + *a_i_*_+1_ *b_i_*_+1_) + *a_i_b_i_*_+1_ + *b_i_ a_i_*_+1_) is the volume of equivalent truncated elliptical cone between the *i th* and *i* + 1 *th* slices.

**Figure 3:**
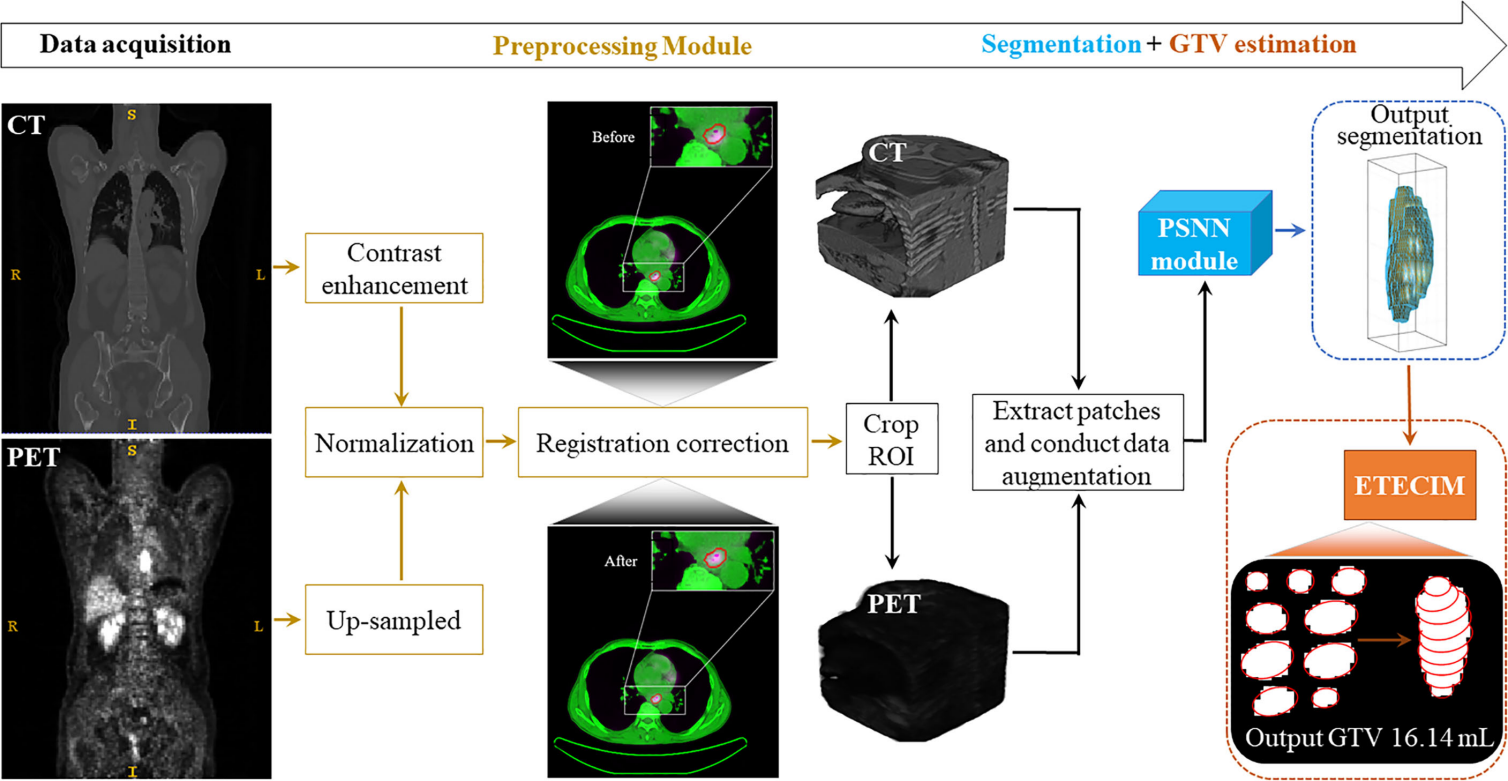
Overview. The whole GTV definition process for ESCC patient includes four stages: data acquisition, data preprocessing, segmentation, and GTV estimation.

To sum up, we provide an overview to display the whole GTV definition process for ESCC patient, as shown in **Figure 3**.

### Discussion Between the RECIST (Version 1.1) and the Proposed ETECIM

In the year 2009, Eisenhauer et al. published the new *Response Evaluation Criteria in Solid Tumors* (RECIST, version 1.1), whose main contents include a standard approach to solid tumor measurement (29). The RECIST guideline defines that, at baseline, measurable tumor lesions must be accurately measured in at least one dimension (longest diameter) with a minimum size of 10 mm for CT scan with slice thickness no greater than 5 mm. For target lesion less than 10 mm (too small to measure), a default measurement of 5 mm should be recorded if the lesion is still present. Besides, the RECIST evaluation also states that using software tools to calculate the maximal diameter for a perimeter of a tumor lesion may even reduce variability. From this perspective, for esophageal tumor, this work proposes the ETECIM to refine the measurement of EC tumor. Further, the estimation of longest and shortest diameters deduces the volumetric assessment, which has been demonstrated as an important prognostic determinant for ESCC patients (9, 10). Therefore, the proposed software algorithm ETECIM refines the measurement of esophageal tumor relative to the RECIST guideline.

### Evaluation Parameters

For the evaluation of segmentation performance, the Dice similarity coefficient (DSC), Hausdorff distance (HD), and mean surface distance (MSD) are used. DSC measures the spatial overlap between the predicted lesion and ground truth (30). HD and MSD respectively measure the maximum distance and the agreement between the predicted and ground truth contours (31). According to the predicted and ground truth tumor, the true positives (TP), false positives (FP), false negatives (FN), predicted contour (*P*), and ground truth contour (*G*) can be calculated. Then the DSC, HD, and MSD are defined,


(10) $$DSC=\frac{2TP}{2TP+FP+FN},$$

(11) $$\text{HD}(P,G)=\max\left\{\underset{p\in P}{\max}\underset{g\in G}{\min}\;d(p,g),\underset{g\in G}{\max}\underset{p\in P}{\min}\;d(p,g)\right\},$$

(12) $$\text{MSD}(P,G)=\frac{1}{2}(\frac{1}{\left|P\right|}\sum_{p\in P}\underset{g\in G}{\min}\;d(p,g)+\frac{1}{\left|G\right|}\sum_{g\in G}\underset{p\in P}{\min}\;d(g,p)+),$$

where *d*(*p*, *g*) denotes the Euclidean distance between surface mesh points *p* and *g*, |*P*| and |*G*| denote the total voxel number of contours *P* and *G* respectively. DSC takes value in [0, 1], and the closer to 1 means larger spatial overlap between the predicted lesion and ground truth. Both the HD and MSD values are greater than or equal to 0, the closer to 0 denotes better segmentation performance.

For the comparison of predicted and ground truth GTVs, conformity index (CI), degree of inclusion (DI), and motion vector (MV) are used. Thereinto, CI and DI assess the spatial relationship, and MV measures the positional change (12, 13, 32). The definitions of CI and DI between volumes A and B are as follows,


(13) $$CI=\frac{A\cap B}{A\cup B},$$

(14) $$DI(A\;in\;B)=\frac{A\cap B}{A},DI(B\;in\;A)=\frac{A\cap B}{B}.$$

CI takes value from 0 to 1, and the value of 1 means that A and B are in complete agreement. For DI, if volume B is the reference for standard volume, and treatment planning is based on volume A, then [1-DI (A in B)] of volume A will be unnecessarily irradiated and [1-DI (B in A)] of volume B will be the missing irradiation part (13). For the calculation of MV, the centers of mass (COM) for volume A and B should be first measured. Afterwards, the displacement of COM for volume A and B in x (left-right (LR)), y (anterior–posterior (AP)) and z (cranial–caudal (CC)) directions will be obtained. Last, MV is calculated as,


(15) $$MV=\sqrt[{2}]{LR^{2}+AP^{2}+CC^{2}}.$$

### Statistical Tests

Statistical analyses are performed using the software package of IBM SPSS Statistics 20.0. Pearson’s correlation is performed to assess the degree of associations between the predicted and ground truth GTVs. The paired sample Student’s t-test is employed for the comparison of GTVs and DIs. One sample t-test is conducted for the LR, AP, and CC. The descriptive statistics are presented in the way of mean ± standard deviation (M ± SD). *P*-values lower than 0.05 are considered to be statistically significant.

## Results

### Visual Comparison of the Predicted and Ground Truth Contours

**Figure 4:**
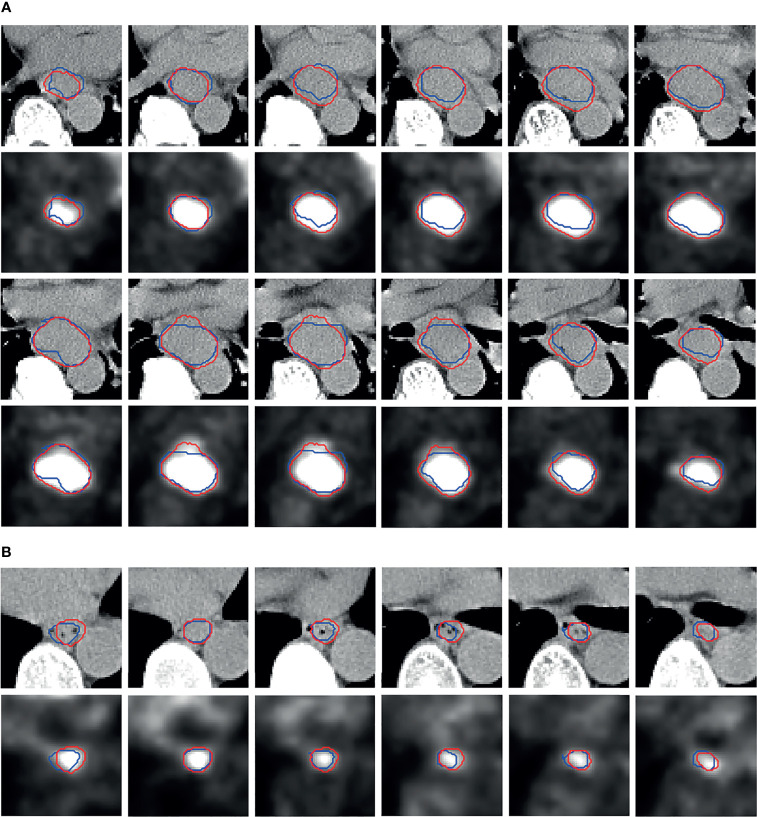
Segmentation visual results. The more slices in patients **(A)** denote larger tumor than **(B)**. The red contours are the predicted results by PSNN, and the blue contours represent the ground truth.

By using the SOTA esophageal GTV segmentation deep neural model PSNN, we report the 4-fold cross-validation results for DSC, HD, and MSD as 0.72 ± 0.02, 11.87 ± 4.20 mm, and 2.43 ± 0.60 mm (M ± SD) respectively. The segmentation visual results of two patients are shown in **Figure 4**.

We can observe that, as a whole, the predicted red contours have good agreement with the blue ground truth contours. Although there are slight biases between the predicted and ground truth lesions, some predicted red contours can enclose the hot areas better compared to the blue ground truth contours in the PET images.

### Differences in GTV

**Figure 5:**
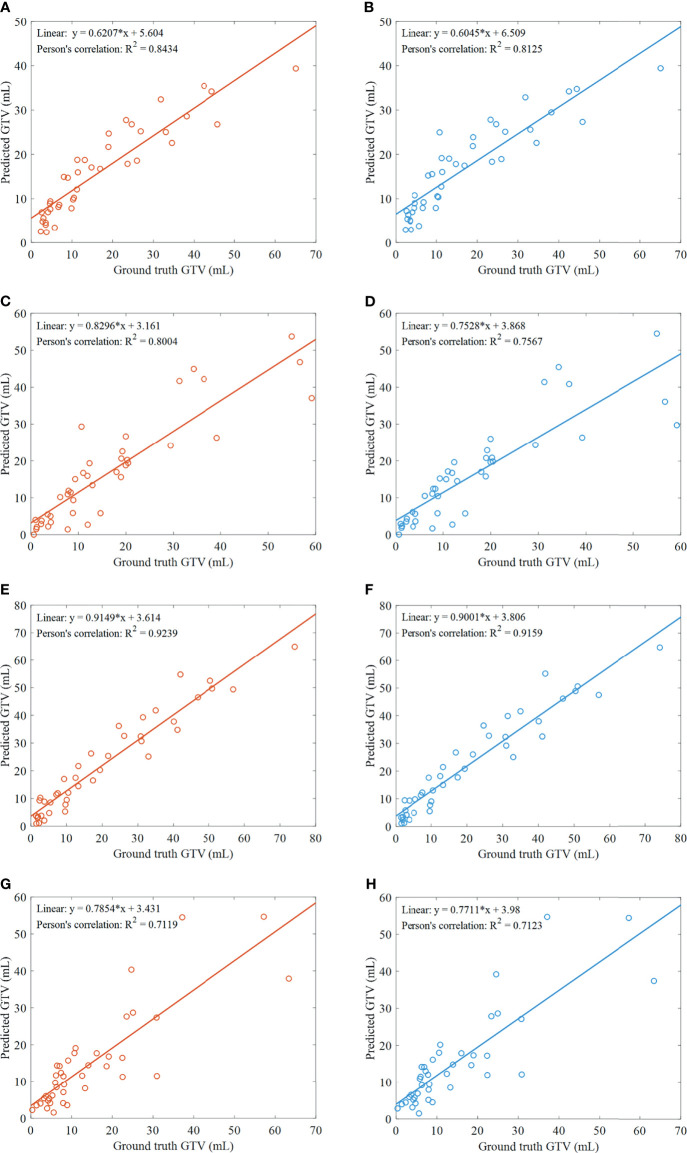
Results of GTV assessment. **(A)** Scatter plot and correlation between the predicted GTVs by ETECIM and manual ground truth GTVs, for the first fold cross-validation. **(B)** Scatter plot and correlation between the predicted GTVs by voxel summation method and manual ground truth GTVs, for the first fold cross-validation. For the same reason, **(C, D)** are results for the second fold cross-validation. **(E, F)** are results for the third fold cross-validation. **(G, H)** are results for the fourth fold cross-validation.

Pearson’s correlation is performed to assess the degree of associations between the ground truth and predicted GTVs by ETECIM. For comparison, Pearson’s correlation is also performed between the ground truth and predicted GTVs by voxel summation method. Results are shown in **Figure 5**. The obtained decision coefficients R^2^ by ETECIM are 0.8434, 0.8004, 0.9239, and 0.7119 for each fold cross-validation, whereas R^2^ by voxel summation method are 0.8125, 0.7567, 0.9159, and 0.7123 for each fold cross-validation. The comparison results indicate that the proposed ETECIM is more accurate than the commonly used voxel summation method to estimate the GTV values.

Further, we conduct the paired sample Student’s t-test for assessing the difference between the predicted GTVs by ETECIM and ground truth GTVs. For the first fold cross-validation, no significant difference is found (*t* = 0.036, *p* = 0.971). For the second fold cross-validation, no significant difference is found (*t* = 0.347, *p* = 0.731). For the third fold cross-validation, there is a significant difference (*t* = 2.388, *p* = 0.022). For the fourth fold cross-validation, no significant difference is found (*t* = 0.326, *p* = 0.746). Though there is a significant difference for the third fold cross-validation, when gathering the predicted GTVs by ETECIM and the ground truth GTVs for all fold cross-validations to conduct the paired sample Student’s t-test, there is no significant difference (*t* = 1.193, *p* = 0.235). Hence, these results indicate that the predicted GTVs by ETECIM are reliable. Besides, **Figures 5A, C, E, G** show that there are linear correlations between the ground truth and predicted GTVs. Hence, according to the corresponding fitted functions, we can reversely solve the ground truth GTV out if giving the predicted GTV value.

### CI and Differences in DI

Using 4-fold cross-validation for evaluation, we report the M ± SD of CI as 0.60 ± 0.16, median CI as 0.63, lower quartile of CI as 0.52, and upper quartile of CI as 0.70, respectively. DIs between the predicted esophageal tumor and ground truth are shown in **Table 1**. The M ± SD of DI (PreT in GroT) and DI (GroT in PreT) are 0.72 ± 0.18, and 0.78 ± 0.20 respectively. There is a significant difference between the DI (PreT in GroT) and DI (GroT in PreT), with the former less than the latter (*t* = −2.636*, p* = 0.009). In reverse, 1 − DI (PreT in GroT) is significantly greater than 1 − DI (GroT in PreT) (*t* = 2.636*, p* = 0.009).

**Table 1:** DI between the predicted and ground truth esophageal tumors.

Pairs		Lower quartile	Median	Upper quartile	M ± SD	*t, p*
Pair 1	DI (PreT^a^ in GroT^b^)	0.63	0.75	0.86	0.72 ± 0.18	−2.636, 0.009
	DI (GroT in PreT)	0.71	0.85	0.92	0.78 ± 0.20	
Pair 2	1- DI (PreT in GroT)	0.14	0.25	0.37	0.28 ± 0.18	2.636, 0.009
	1- DI (GroT in PreT)	0.08	0.15	0.29	0.22 ± 0.20	

a PreT denotes the predicted esophageal tumor by PSNN net. ^b^GroT represents the manual ground truth tumor.

### Differences in Position

One sample t-test is conducted on LR, AP, and CC respectively, with a test value of 0. **Table 2** lists the detailed results of one sample t-test. No significant difference is found, except for CC of the first fold cross-validation (*t* = −2.031, *p* = 0.049) and CC of the fourth fold cross-validation (*t* = 3.333, *p* = 0.002). But for the whole 4-fold cross-validation, no significant differences are found in LR (*t* = 0.102, *p* = 0.919), AP (*t* = 0.221, *p* = 0.826), and CC (*t* = 0.569, *p* = 0.57) directions. As for MV, we get the M ± SD, lower quartile, median, and upper quartile as 1.90 ± 2.4, 0.93, 1.30, and 1.97 mm respectively. The SD is a little big because of a several not accurate segmentation masks.

**Table 2:** Position differences between the predicted and ground truth tumors.

4-fold cross-validation	LR	AP	CC
First fold	*t* = −0.727, *p* = 0.472	*t* = −0.066, *p* = 0.947	*t* = −2.031, *p* = 0.049
Second fold	*t* = 0.912, *p* = 0.367	*t* = 0.659, *p* = 0.514	*t* = 1.537, *p* = 0.132
Third fold	*t* = 0.534, *p* = 0.596	*t* = 0.514, *p* = 0.610	*t* = 1.512, *p* = 0.138
Fourth fold	*t* = −1.373, *p* = 0.177	*t* = −1.021, *p* = 0.314	*t* = 3.333, *p* = 0.002
Overall	*t* = 0.102, *p* = 0.919	*t* = 0.221, *p* = 0.826	*t* = 0.569, *p* = 0.57

## Discussion

^18F^FDG PET/CT-guided precise diagnosis, treatment and prognosis rely on the accurate definition of esophageal carcinoma. The current manual definition manner is time consuming, operator dependent and fluctuant, indirectly leading to the problem of oversized IF. Thus, how to precisely and intelligently define the lesion area from the obtained medical images has become an urgent issue. Some studies have explored the fully auto-delineation of esophageal carcinoma by using deep learning-based methods. However, the estimation of GTV values and the relevant evaluation are missed. In the present work, we take the automatic segmentation task one step further, that is to say, we extra estimate the GTV of ESCC and assess whether the intelligent definition method is potentially applicable to help clinicians to further determine precise IF.

We first employ the SOTA esophageal GTV segmentation deep model PSNN to conduct the automatic segmentation task, and obtained the DSC, HD, and MSD as 0.72 ± 0.02, 11.87 ± 4.20 mm, and 2.43 ± 0.60 mm respectively. From the visual results (**Figure 4**), despite the existing slight biases between the predicted and ground truth lesions, good agreement is found as a whole, and some predicted red contours are more accurate to enclose the hot areas in PET images. Based on the segmentation results by PSNN, we next propose the ETECIM to estimate the GTV values. To provide reliable references for the potential clinical application, statistical analyses are conducted to evaluate the differences between predicted results and ground truth.

Pearson’s correlation is performed, and we get correlation coefficients of 0.8434, 0.8004, 0.9239, and 0.7119 for each fold cross-validation between the ground truth and predicted GTVs by the proposed ETECIM (**Figures 5A, C, E, G**). For comparison, **Figures 5B, D, F, H** illustrate the correlation between the ground truth and predicted GTVs by the voxel summation method. Results demonstrate that the proposed ETECIM for GTV estimation is more accurate and closer to the ground truth GTV than the voxel summation method. When the paired sample Student’s t-test was conducted, no significant difference was found (*t* = 1.193, *p* = 0.235) between the predicted GTVs by ETECIM and the ground truth GTVs. Besides, the good linear correlation can derive the true GTV value.

For CI and DI, which synthetically reflect the geometrical differences between the predicted tumor and ground truth, we report the median CI as 0.63, the M ± SD of DI (PreT in GroT) and DI (GroT in PreT) are 0.72 ± 0.18, and 0.78 ± 0.20 respectively. According to the study of Shi et al. (13), the median CI approximated to 0.7 denotes that the predicted and ground truth tumor corresponds well. For DI (PreT in GroT) and DI (GroT in PreT), a significant difference is found (*t* = −2.636, *p* = 0.009). DI (GroT in PreT) is larger than DI (PreT in GroT), thus 1 − DI (GroT in PreT) is significantly less than 1 − DI (PreT in GroT) (*t* = 2.636, *p* = 0.009). This indicates that if the radiotherapy is based on the predicted tumor, the possibility of missing the lesion is low. In the meanwhile, there is a little unnecessary irradiation to the surrounding tissue. But in practice, GTV is contained in clinical target volume, which describes the extent of microscopic and un-imageable tumor spread (4). Clinical target volume is obtained *via* expanding and measuring the adjacent sub-clinical disease margins around GTV (Defined in China: GTV + 3 cm margins in the esophageal long axis superiorly and inferiorly, and GTV + 0.5 cm margins in the cross section to encompass potential submucosal invasions) (9). Therefore, the unnecessary little irradiation to surrounding tissue is acceptable. As for how much irradiation is suitable and how many margins need to be added on the basis of predicted GTV, detailed clinical treatment data are needed to study these problems.

For differences in position, results of the one sample t-test show that there are no significant differences in LR (*t* = 0.102, *p* = 0.919), AP (*t* = 0.221, *p* = 0.826), and CC (*t* = 0.569, *p* = 0.57) directions, and the obtained MV is small. Hence, these results demonstrate that the segmented masks correspond well with the ground truth.

## Conclusions

In this work, we have assessed the applicability of the artificial intelligence-based method for fully automatic GTV definition of ESCC on 3D ^18^F-FDG PET/CT. The visual segmentation results indicate good agreement between the predicted and ground truth tumors. The quantitative results demonstrate that the proposed ETECIM is more accurate than the most commonly used voxel addition method to estimate GTV values. Statistical analyses demonstrate that radiotherapy planning based on the predicted tumor is potentially feasible, and radiologists can take artificial intelligence method to define GTV of ESCC patients, as an efficient auxiliary means to refine the manual definition to further determine a more precise IF. In the future, more studies based on the specific clinical treatment data need to be conducted to validate and push this application forward.

## Data Availability Statement

The raw data supporting the conclusions of this article will be made available by the authors, without undue reservation.

## Ethics Statement

This retrospective study was approved by the medical ethics committee of our institution. Informed consent was obtained from all individual participants included in the study.

## Author Contributions

YY conception, design, methodology, data assembly, software, writing (original draft). NL conceptualization, design, data collection and delineation, validation, writing (review and editing). HS writing (review and editing). DB writing (review and editing). XL supervision, conceptualization, writing (review and editing). SS supervision, data collection and delineation. DT supervision, writing (review and editing). All authors listed have made a substantial, direct, and intellectual contribution to the work and approved it for publication.

## Funding

This work was supported by the National Natural Science Foundations of China (81771861 and 61871263), the Program of Shanghai Academic Research Leader (19XD1400500), and the Fudan University Shanghai Cancer Center Foundation (YJ201807).

## Conflict of Interest

The authors declare that the research was conducted in the absence of any commercial or financial relationships that could be construed as a potential conflict of interest.

## Publisher’s Note

All claims expressed in this article are solely those of the authors and do not necessarily represent those of their affiliated organizations, or those of the publisher, the editors and the reviewers. Any product that may be evaluated in this article, or claim that may be made by its manufacturer, is not guaranteed or endorsed by the publisher.
